# Further Explorations of the Facing Bias in Biological Motion Perception: Perspective Cues, Observer Sex, and Response Times

**DOI:** 10.1371/journal.pone.0056978

**Published:** 2013-02-27

**Authors:** Ben Schouten, Alex Davila, Karl Verfaillie

**Affiliations:** Laboratory of Experimental Psychology, KU Leuven, Leuven, Belgium; University of Alberta, Canada

## Abstract

The human visual system has evolved to be highly sensitive to visual information about other persons and their movements as is illustrated by the effortless perception of point-light figures or ‘biological motion’. When presented orthographically, a point-light walker is interpreted in two anatomically plausible ways: As ‘facing the viewer’ or as ‘facing away’ from the viewer. However, human observers show a ‘facing bias’: They perceive such a point-light walker as facing towards them in about 70-80% of the cases. In studies exploring the role of social and biological relevance as a possible account for the facing bias, we found a ‘figure gender effect’: Male point-light figures elicit a stronger facing bias than female point-light figures. Moreover, we also found an ‘observer gender effect’: The ‘figure gender effect’ was stronger for male than for female observers. In the present study we presented to 11 males and 11 females point-light walkers of which, very subtly, the perspective information was manipulated by modifying the earlier reported ‘perspective technique’. Proportions of ‘facing the viewer’ responses and reaction times were recorded. Results show that human observers, even in the absence of local shape or size cues, easily pick up on perspective cues, confirming recent demonstrations of high visual sensitivity to cues on whether another person is potentially approaching. We also found a consistent difference in how male and female observers respond to stimulus variations (figure gender or perspective cues) that cause variations in the perceived in-depth orientation of a point-light walker. Thus, the ‘figure gender effect’ is possibly caused by changes in the relative locations and motions of the dots that the perceptual system tends to interpret as perspective cues. Third, reaction time measures confirmed the existence of the facing bias and recent research showing faster detection of approaching than receding biological motion.

## Introduction

The human visual system is extremely well adapted to decode visual information about another person and his or her movements, particularly when this information is socially or behaviorally relevant. For example, when the visual information is reduced to a video display of a group of point-lights representing the main joints of that person, human observers still easily infer the gender [1]–[4], identity [5]–[7], age [8], emotional state [9]–[14], and intention [15], [16] of the actor. Ever since so-called ‘point-light figures’ were introduced by Johansson [17], they have proven to be a well suited stimulus to study the visual perception of biological motion.

One property of point-light figures that only relatively recently gained scientific interest is their in-depth interpretation. Vanrie, Dekeyser, and Verfaillie [18] demonstrated that, when presented orthographically on a 2D screen, a point-light walker is interpreted in two anatomically plausible ways: The figure is either perceived as ‘facing the viewer’ (FTV) or as ‘facing away’ (FA) from the viewer. Interestingly, besides the bistable nature of this stimulus Vanrie et al. [18] found evidence for a preferred interpretation. That is, when presented with an in principle ambiguous point-light walker, human observers perceive the point-light walker as facing towards them in about 70–80% of the cases. Subsequent studies [19]–[21] confirmed this finding, however, the causes of this ‘facing bias’ remain unclear.

One possible account of the facing bias could be based on social and biological relevance. Indeed, in most cases another person facing the observer probably is socially more relevant than a person facing away. The perceptual system might therefore take into account the potential cost of misinterpreting the actions and intentions of others: Expecting someone to approach who is actually retreating might be less costly than misinterpreting an approach for a retreat.

Exploring the role of social and biological relevance Brooks, Schouten, Troje, Verfaillie, Blanke, and van der Zwan [1] investigated the effect of the perceived gender of an ambiguous point-light figure on its perceived in-depth orientation. In the study of Vanrie et al. [18] only male walkers were shown. However, if social and biological relevance indeed affect perceptual in-depth organization then the cost of misinterpreting actions could be different for the perception of male conspecifics than for the perception of female conspecifics. For instance, an approaching male might be perceived more threatening than an approaching female. Consequently, male and female ambiguous point-light figures might elicit different degrees of the facing bias. Using depth-ambiguous point-light figures in frontal/back view, and varying gender on a continuum from extremely male to extremely female (see [4]), Brooks et al. collected both gender ratings and in-depth orientation ratings and found a strong correlation between perceived gender and perceived orientation of ambiguous point-light walkers. Consistent with the facing bias found by Vanrie et al., figures perceived as male were generally perceived as facing the viewer. The more the point-light figures were perceived as female, however, the more the figures were perceived as facing away from the viewer.

Schouten, Troje, Brooks, van der Zwan, and Verfaillie [22] further investigated the effect of figure gender and the role of observer sex in a larger sample of male and female observers. While weaker, Schouten et al. again observed a significant figure gender effect: Male figures elicited a stronger facing bias than female figures did. But more importantly, Schouten et al. also observed a small but significant interaction between stimulus gender and observer sex: The facing bias for male point-light walkers appeared to be stronger for male observers than for female observers.

Finally, Schouten, Troje, and Verfaillie [23] demonstrated that the relation between perceived gender and perceived in-depth orientation as observed in Brooks et al. [1] and Schouten et al. [22] is not directly causal. Structural and kinematic stimulus changes that induced comparable changes in perceived gender were found to elicit opposite changes in perceived in-depth orientation. Thus, stimulus properties, irrespective of the perceived gender they elicit, were found to play a crucial role in biasing the in-depth perception of depth-ambiguous point-light walkers. Patterns of FTV responses in Schouten et al. [23] that most resembled the response patterns that were earlier observed in Brooks et al. [1] and Schouten et al. [22] were the patterns of FTV responses observed for point-light figures of which the gender only varied based on structural information, suggesting that structural stimulus changes underlie the earlier observed figure gender effect. Why precisely these structural stimulus changes lead to such strong changes in perceived in-depth orientation is not clear. One possibility that was mentioned in Schouten et al. [22] was that some of the geometric features that distinguish male and female point-light walkers may be misinterpreted by the visual system as perspective distortions. Yet, so far, this ‘perspective account’ has never been explored. Therefore, it would be interesting to test whether observer sex effects in the in-depth perception of point-light figures as observed by Schouten et al. [22] can also be observed for stimuli that do not vary in depicted gender but vary only in the amount of perspective information they carry. Demonstrating an observer sex effect for stimuli that only vary in perspective information would be the first important and necessary step in further exploring the ‘perspective account’. The primary goal of the present study was to test whether or not we can reproduce an observer sex effect as found in Schouten et al. [22] with a gender neutral point-light figure of which only the perspective cues that it carries are manipulated.

Schouten and Verfaillie [24] recently demonstrated that the perceived in-depth orientation of point-light figures can be gradually manipulated by introducing perspective cues. This technique allows to manipulate the perceived in-depth orientation of a point-light walker from convincingly facing the viewer to convincingly facing away and to determine the point of subjective ambiguity (PSA). The PSA indicates the stimulus that by a particular observer is perceived as perfectly ambiguous, that is, perceived as facing the viewer in half of the cases and as facing away in the other half of the cases. The perspective technique is thus perfectly suited for the purpose of the present study. However, one could argue that the perspective technique described by Schouten and Verfaillie [24] has a limitation because the distortions at the projection plane involve distortions of the shape and size of the point-light dots. As changes in the shape and size of the dots may act as local cues, it is possible, in principle, that observers base their responses on these additional local cues. To exclude this possibility, in the present study we employed the perspective technique but removed all local shape and size cues. The secondary goal of the present study was thus to verify whether the perspective technique [24] still convincingly drives perceived in-depth orientation of a point-light walker when local shape and size cues are removed and perspective cues are confined to very subtle changes in the positions and motions of the point-light dots.

Finally, in most of the experiments on the facing bias in biological motion perception [1], [22]–[25] proportions of FTV responses were recorded. The average proportion of FTV responses of a group of observers viewing a particular point-light stimulus gives a good indication about the ambiguity of the stimulus. Indeed, if the proportion of FTV responses is close to 0.50 the stimulus can be considered as perceptually ambiguous. If, for example, the proportion of FTV responses for a particular point-light figure is above 0.80 this indicates that the stimulus more easily stabilizes to a percept of a walker facing the viewer. In addition to proportions of FTV responses reaction times may be another potentially informative measure on the ambiguity of the in-depth percept [26]. As far as we know, response times were never used to explore the in-depth ambiguity resolution of point-light figures. The third goal of the present study is to explore how response times relate to proportions of FTV responses.

## Materials and Methods

### Participants

22 observers (11 females and 11 males) participated in this experiment. All observers had normal or corrected to normal vision, had provided informed written consent, and were naïve to the purpose of the experiment.

### Ethics Statement

The study was approved by the Ethical Committee of the Faculty of Psychology and Educational Sciences of the KU Leuven and in accordance with the ethical standards laid down in the 1964 Declaration of Helsinki.

### Stimuli and apparatus

The point-light stimulus used in the present study was the gender-neutral walker (0 SD) derived from the continuum described in detail by Troje [4]. The amount of perspective information that was provided by this stimulus was manipulated according to the perspective technique described in Schouten and Verfaillie [24]. However, one important aspect of this technique was adapted. Whereas in Schouten and Verfaillie [24] the dots changed in shape and size depending on the field of view angle of the perspective projection, here first the 2D positions of the dots were computed. Then, dots of a constant shape and size were placed on these positions. This resulted in a set of point-light walkers of which only the relative positions and motions of the dots were informative on the point-light figure’s in-depth orientation. As in Schouten and Verfaillie [24], the perspective cues were gradually manipulated from absent (orthographic projection) to very strong. Note that here, as in Experiment 2 of Brooks et al. [1], for half of the figures containing perspective cues, perspective was added to make the figures appear more facing the viewer (negative field of view angle values). In the other half of the figures perspective cues signaled a walker facing away (positive field of view angle values). In total, we used 13 levels of perspective information that were composed of one orthographic projection and 12 distance (D) manipulations of the convergence point, six on the front side and six on the back side of the walker, corresponding to 16, 8, 6, 4, 3, and 2 times the height of the walker. This resulted in field of view angles of 14, 28, 37, 53, 67, and 90 degrees, respectively. As in Schouten and Verfaillie [24], the height of the point-light figures was adjusted according to the viewing distance and subtended about seven degrees of visual angle. Hence, the height of the screen (four times the height of the walker) subtended about 28 degrees. Each dot subtended about 15 arc mins. The start position of the animation cycle (117 frames/cycle) was randomized across trials.

### Procedure

Observers were seated in a dimly lit and sound attenuated room. Viewing distance was 57cm from a CRT monitor (refresh rate = 120 Hz). On each trial, after presentation of a 500 ms fixation cross, observers were shown a depth-ambiguous point-light walker and had to indicate by a key-press (arrow down for FTV, arrow up for FA) whether the walker was perceived as oriented towards or away from them. Reaction times were recorded. Observers were instructed to respond according to their own subjective experience and it was stressed that an equal distribution of the two response alternatives was not necessary. The point-light walker was presented until response. Observers were encouraged to respond as fast as possible as soon as they experienced a stable in-depth percept. After instructions were given, observers completed a practice block (random selection of all possible conditions) in which they were familiarized with the stimuli and the task. Then it was checked again whether they had understood the task and the experiment commenced. In total, each observer completed 520 trials (13 levels * 40 repetitions). Trial order was randomized and trials were divided in 13 blocks of 40 trials each. In total, the experiment lasted about 35 minutes.

## Results

First, as in Schouten and Verfaillie [24], we verified whether the proportions of FTV responses as a function of the field of view angle were well fit with a cumulative Gaussian. Deviance of the observations to the cumulative Gaussian fit [27], [28] was not significantly higher than simulated deviance (10,000 Monte Carlo simulations) for 19 out of 22 observers (all *p* values > 0.05, except for three observers). Slopes varied between −0.021 and 0.0059 with a mean of −0.0073 (SD = 0.0064). The unit of a slope of a line is defined as the change in y value divided by the change in x value (dependent and independent variables, respectively). In the present data the y value is the proportion of facing the viewer (FTV) responses and the x value is the field of view angle (FVA). So, the unit of the indicated slopes is the change in proportion of FTV responses divided by the change in FVA. Mean PSA values varied between −13.28° and 357.20° with a mean of 62.57°. Compared to what was observed in Schouten and Verfaillie [24] (mean PSA = 40°), here the PSA seems to converge to a higher field of view angle. However, this value is strongly affected by outliers. Some observers had a very strong facing bias leading to extremely high PSA values. Note that extreme PSA values (e.g., 357.20°) have no meaningful interpretation. In fact, for these individuals none of the perspective distortions are strong enough to induce the ‘facing away’ interpretation. When a single cumulative Gaussian was fit to all data (in the fitting procedure of Wichmann and Hill [27], [28] values are weighted according to their reliability), the mean PSA converges to 47.30° which is more in the range of the mean PSA that was found in Schouten and Verfaillie [24]. Based on these data we can conclude that also with the shape and size of the dots kept constant across perspective levels, the perspective technique works well to manipulate the perceived in-depth orientation and to determine the PSA.

**Figure 1:**
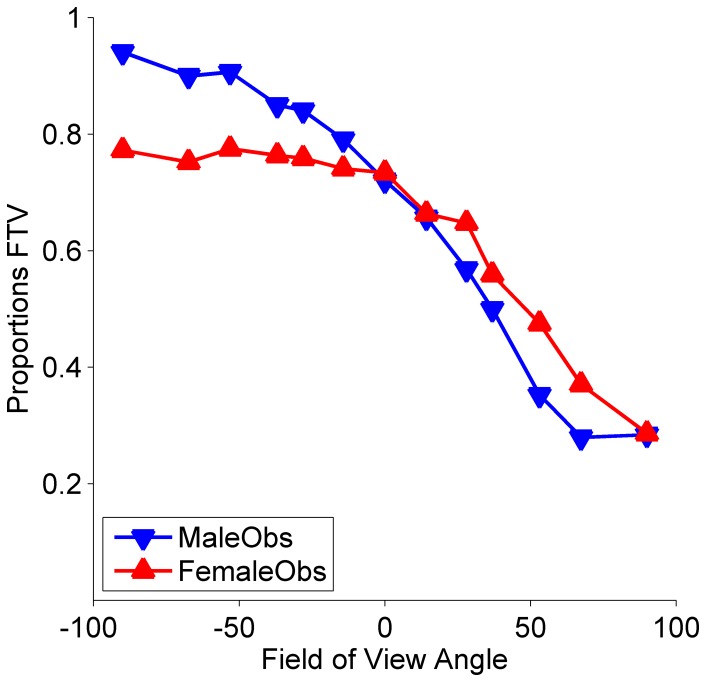
Proportions of ‘facing the viewer’ (FTV) responses as a function of field of view angle. Data of 11 females and 11 males. The more negative the field of view angle (perspective level) is, the stronger is the perspective cue that the point-light figure is facing away from the viewer. The higher the field of view angle is, the stronger the perspective cue is that the point-light figure is facing towards the viewers. Local shape and size of the dots of the point-light figures were kept constant across perspective levels. Even without local shape and size cues observers seem to easily pick up on the remaining position and motion cues: Mean proportions of FTV responses gradually drop as a function of field of view angle. The effect is stronger for male (blue triangles pointing downwards) than for female observers (red triangles pointing upwards). Female observers respond differently to the same stimuli than male observers. This pattern of results is consistent with the earlier observed ‘observer sex effect’ [22].

Second, to compare the present results with the results of Schouten et al. [22] we performed a repeated measurement ANOVA on the probit transformed proportions with the field of view angle as a within subject variable and observer sex as a between subjects variable. As expected based on what was described in the previous paragraph we found a significant effect of field of view angle on the perceived in-depth orientation (F(20,240) = 30.18, p<0.001). Although observer sex itself did not show a main effect on the perceived in-depth orientation (F(1,20) = 0.71, p = 0.41), we found a significant interaction between field of view angle and observer sex (F(12,240) = 2.44, p<0.01). In this respect, the present results are quite similar to what was observed in Schouten et al. [22]. In Schouten et al. [22], stimulus gender had a stronger effect on male observers than on female observers. Here, the perspective projection had a stronger effect on male observers than on female observers. In Figure 1 we plot the proportions of FTV responses as a function of field of view angle for male and female observers separately. From the plot it is clear that the changes in proportions of FTV responses as a function of field of view angle were stronger for male than for female observers. This was also confirmed by a significant difference in the slope parameters of two cumulative Gaussians, one fitted to the male (slope = −0.0068, 95% C.I. = [−0.0075, −0.0063]) and one fitted to the female observer data (slope = −0.0034, 95% C.I. = [−0.0038, −0.0030]). In addition, note that one of the female observers had a positive instead of a negative slope. Note that this does not mean that this subject could not relate to the question (towards or away). On the contrary, the Monte Carlo check indicated that the data of this subject were quite well fit by a cumulative Gaussian curve. The sign of the slope, however, differed from the other subjects. After the experiment for this subject we explicitly checked and confirmed that response keys were not confused. While it is outside the scope of the present manuscript to discuss in detail the possible causes, earlier experiments in our lab (Schouten, Troje, & Verfaillie, 2011) [23] have indicated that (stable) inverse relations of proportions of FTV responses to the manipulated variable are possible and are linked to relying on alternative cues in the point-light figure. Each of the three earlier mentioned observers for which the deviance exceeded the simulated deviance (10,000 Monte Carlo simulations) were female. Female observers thus respond quite differently to the same stimuli than male observers.

**Figure 2:**
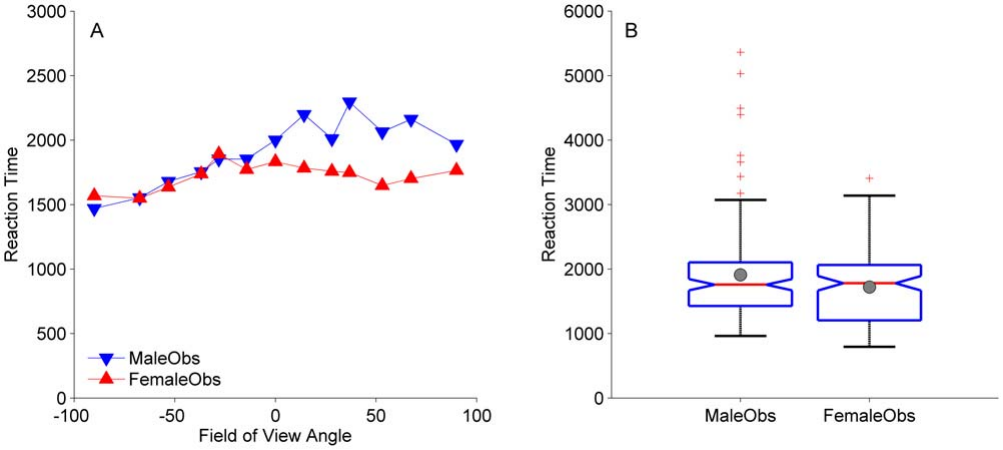
Reaction time as a function of perspective cue and boxplots for male and female observers. Data of 11 females and 11 males. **A**. Mean reaction times generally increase as a function of field of view angle. The clearer the point-light figure is facing the viewer (strongly negative field of view angles), the quicker observers are to respond. The increase in reaction time as a function of field of view angle saturates or even reverses to a slight decrease for walkers carrying perspective cues that convincingly signal the walker to be ‘facing away’. It appears as if female observers have shorter response times than male observers, mainly for figures containing ‘facing away’ perspective cues. **B**. The box plots, however, clarify that the somewhat larger mean for male compared to female observers (grey circles) resulted from outliers (red crosses) in the data of the male observers. The red horizontal lines in the middle of the boxes indicate the median. The bottom of the box indicates the 25 th percentile. The top of the box represents the 75 th percentile. The T-bars or whiskers extend to 1.5 times the height of the box or, if no observation has a value in that range, to the minimum or maximum values. The notches of the box plots (triangles pointing inwards), indicating the 95% confidence interval around the median, overlap: Median reaction times of male and female observers do not differ.

Third, we explored response times and their relation to the proportions of FTV responses. In Figure 2 we plotted the reaction times as a function of field of view angle for male and female observers, separately. Mean response time of all responses was 1818 ms. To analyse response times, we first fit for each observer a second-order polynomial to the response times as a function of field of view angle. We then analyzed the values of the curvature, the slope, and the constant with (paired) t-tests. From Figure 2A it appears as if female observers have shorter response times than male observers, mainly for figures containing ‘facing away’ perspective cues. However, the difference between the constants of female observers (1780 ms) and male observers (2012 ms) was not significant (*t*(10) = 1.01, *p* = 0.34). The box plots in Figure 2B clarify that the somewhat larger mean for male compared to female observers (grey circles) resulted from outliers (red crosses) in the data of the male observers. The notches of the box plots (triangles pointing inwards) that indicate the 95% confidence interval around the median are overlapping, showing that median reaction times of male and female observers did not differ. Curvature (*t*(10) = −0.58, *p* = 0.57) and slope (*t*(10) = 1.5235, *p* = 0.16) parameters did also not differ significantly between female and male observers. However, response times as a function of field of view angle across all observers showed a significantly positive slope (*t*(21) = 2.16, *p* = 0.043) and a significantly negative curvature (*t*(21) = −2.16, *p* = 0.043). A repeated measurement ANOVA (with field of view angle as a within subject and observer sex as a between subjects factor) on the response times showed that only the effect of field of view angle was significant (*F*(12,240) = 3.71, *p*<0.001). The effect of observer sex (*F*(1,20) = 0.64, *p* = 0.43) and the (outlier driven) trend of the interaction of observer sex with the field of view angle (*F*(12,240) = 1.69, *p* = 0.069) were not significant.

Finally, we checked whether the response times correlated with the uncertainty reflected by FTV responses. It could be expected, for example, that the closer the proportion of FTV responses are to 0.50, the longer the response time is, because uncertainty about the perceived in-depth orientation of such point-light figures is highest. In contrast, the more the proportions of FTV responses differ from 0.50, the less uncertain an observer is about the perceived in-depth orientation. This was tested by probit transforming the proportions of FTV responses to z-scores. The probit link transforms proportions of FTV responses close to 0.50 to values near zero and proportions of FTV responses above and below 0.50 to positive and negative values, respectively. The absolute value of the z-scores could then be conceived as a measure of certainty about the response. If our expectations were right, these values should negatively correlate with response times. Across all conditions and observers the Spearman correlation between response times and the absolute value of z-scores of FTV responses was indeed significantly negative, *r*(284) = −0.25, *p*<0.001.

## Discussion

In the present study we modified the perspective technique of Schouten and Verfaillie [24]. More specifically, we kept local shape and size of the dots of the point-light figures constant across perspective levels in order to control for the potential effect of local cues in the observers’ estimation of the in-depth orientation of the point-light figure. Results indicate that even without local shape and size cues observers easily pick up on the remaining position and motion cues. This observation confirms that the visual system is extremely sensitive to perspective cues mimicking the subtle changes on the observers’ retina when another person is approaching or retreating. Such sensitivity could of course be of high evolutionary relevance. Recent studies indeed support the idea that the visual system has evolved to be highly tuned to detect approaching biological motion. Sweeny, Haroz, and Whitney [29], for example, demonstrated an increased sensitivity for perceiving approaching headings and a repulsive perceptual effect around the categorical boundary of leftward/rightward motion. Moreover, using a visual search task, Doi and Shinohara [30] recently showed that a stereoscopically presented point-light walker is detected faster when binocular disparity cues are provided that the walker is approaching, compared to when disparity cues indicate that the figure is receding.

Another objective of the present study was to check whether for stimuli that only vary in perspective information an observer gender effect can be found as was observed in Schouten et al. [22]. The results of the present study show that this is indeed the case. That is, we again observed a significant difference between male and female observers in how strongly proportions of FTV responses depend on stimulus variations that cause variations in perceived in-depth orientation. Or, in other words, whereas in Schouten et al. [22] we found an interaction between observer sex and figure gender, in the present study we found an interaction between observer sex and perspective cues. This indicates that differences in response patterns between male and female observers are not specific to stimuli that vary on the gender dimension. The interaction effect between observer sex and figure gender is thus not necessarily linked to perceived gender. This observation is consistent with the findings of Schouten et al. [23] that suggest that not perceived gender but correlated stimulus properties cause the changes in perceived in-depth orientation (also see [31]). But what then precisely underlies the figure gender effect that consistently has been observed [1], [22], [23]? One hypothesis that gains support by the present data is that the figure gender effect is caused by changes in the relative locations and motions of the dots that the perceptual system tends to interpret as perspective cues. We are currently further exploring this ‘perspective hypothesis’.

The question remains as to why perspective cues are differently interpreted by male and female observers. While the present data cannot answer this question it might be relevant to note that also in the auditory domain listener sex differences have been found when it concerns looming and receding sound sources. Indeed, Neuhoff [32] showed that human listeners convincingly perceive rising intensity tones (suggesting a looming sound source) to change in loudness significantly more than falling intensity tones (suggesting a receding sound source) despite an equal amount of intensity change in each condition. Neuhoff, Planisek, and Seifritz [33] examined sex differences in audiospatial perception of sounds that moved toward and away from the listener. Females more than males overestimated arrival time and arrival position of the moving sound source. Grassi [34] found that females more than males overestimated the duration of looming sounds in comparison to receding sounds. Analogues between biases in the auditory domain and the facing bias in biological motion perception have been suggested before [25]. Future research should investigate in more detail whether the facing bias and observer sex effects as observed in the present study and before are the result of evolutionary pressures comparable as the ones that are assumed to underlie biases and observer sex effects in the auditory domain [33].

Finally, in the present study we also explored whether and how reaction times relate to proportions of FTV responses. The data suggest that reaction times to a certain degree capture the same uncertainty/certainty that is assumed to be reflected by proportions of FTV responses. The closer proportions of FTV responses are to 0.50, the slower response times. The further away proportions are from 0.50, the faster observers tend to respond. Interestingly, as suggested by the significant positive slope of response times as a function of field of view angle, observers are faster in responding to figures that carry cues that the figure is facing towards the viewer and are slower in responding to figures that carry ‘facing away’ cues. In other words, observers more quickly reach a stable percept of a walker who is facing towards them than of a walker who is facing away from them, an observation that - for the first time with an alternative measure - confirms the existence of a facing bias in biological motion perception. Note that this observation is also consistent with the recent finding that approaching biological motion is detected faster than receding biological motion [30]. The significant curvature indicates that the increase in reaction time saturates or even reverses to a slight decrease for figures that carry perspective cues that convincingly signal the point-light walker to be ‘facing away’. Hence, as could be expected, uncertainty about the in-depth orientation of those figures in terms of response times seems to drop again.

## Conclusions

In sum, first we showed that human observers easily pick up on perspective cues even in the absence of potential local shape or size cues, confirming a high visual sensitivity to cues that are informative on whether biological motion is potentially looming [29]. Second, there appears to be a consistent difference in how male and female observers respond to stimulus variations (figure gender or perspective cues) that cause variations in the perceived in-depth orientation of a point-light walker. Possibly, the figure gender effect [1], [22], [23] is caused by changes in the relative locations and motions of the dots that the perceptual system tends to interpret as perspective cues. Finally, our reaction time measures confirmed the existence of the facing bias and the finding that approaching biological motion is detected faster than receding biological motion [30].
